# Metal Coordination-Driven Supramolecular Nanozyme as an Effective Colorimetric Biosensor for Neurotransmitters and Organophosphorus Pesticides

**DOI:** 10.3390/bios13020277

**Published:** 2023-02-15

**Authors:** Preeti Bhatt, Manju Solra, Smarak Islam Chaudhury, Subinoy Rana

**Affiliations:** Materials Research Centre, Indian Institute of Science, C.V. Raman Road, Bangalore, Karnataka 560012, India

**Keywords:** nanozyme, biosensing, neurotransmitters, organophosphorus pesticides, supramolecular self-assembly

## Abstract

Analytical methods for detecting neurotransmitters (NTs) and organophosphorus (OP) pesticides with high sensitivity are vitally necessary for the rapid identification of physical, mental, and neurological illnesses, as well as to ensure food safety and safeguard ecosystems. In this work, we developed a supramolecular self-assembled system (SupraZyme) that exhibits multi-enzymatic activity. SupraZyme possesses the ability to show both oxidase and peroxidase-like activity, which has been employed for biosensing. The peroxidase-like activity was used for the detection of catecholamine NTs, epinephrine (EP), and norepinephrine (NE) with a detection limit of 6.3 µM and 1.8 µM, respectively, while the oxidase-like activity was utilized for the detection of organophosphate pesticides. The detection strategy for OP chemicals was based on the inhibition of acetylcholine esterase (AChE) activity: a key enzyme that is responsible for the hydrolysis of acetylthiocholine (ATCh). The corresponding limit of detection of paraoxon-methyl (POM) and methamidophos (MAP) was measured to be 0.48 ppb and 15.8 ppb, respectively. Overall, we report an efficient supramolecular system with multiple enzyme-like activities that provide a versatile toolbox for the construction of sensing platforms for the colorimetric point-of-care detection of both NTs and OP pesticides.

## 1. Introduction

Neurotransmitters (NTs) such as norepinephrine, epinephrine, and acetylcholine are crucial for maintaining both physical and mental health in humans [1]. While norepinephrine is essential for mental illnesses, neurological diseases, and pheochromocytoma, epinephrine is a vital chemical transmitter that modulates blood flow throughout the body [2]. Acetylcholine (ACh) is one of the most significant NT in our body and is in charge of our cholinergic system, including the central and peripheral nervous system [3]. Acetylcholine esterase (AChE) is the enzyme responsible for converting acetylcholine into choline and acetic acid. An imbalance of AChE enzyme concentration is linked to several neurological disorders, including Alzheimer’s disease, dementia, Parkinson’s disease, and schizophrenia, making AChE and ACh significant physiological biomolecules [4]. Certain nerve agents, including carbamates and organophosphorus (OP) chemicals, have anticholinergic characteristics inhibiting AChE, with consequent systemic nervous problems in the body. The monitoring of these NTs is vital for the delivery of quality healthcare, while the quantitative measurement of OP compounds is extremely desirable for reasons of public safety and environmental protection. Several analytical techniques, including high-performance liquid chromatography [5], fluorometric analysis [6,7], and electrochemical methods [8,9], have been developed in recent years for the monitoring of NT levels and the effective detection of pesticides. Even while these approaches can accurately detect their presence, they typically require expensive equipment and laborious sample processing. Therefore, it is crucial for the development of quick, affordable, sensitive, and equipment-free techniques for the precise and uncomplicated detection of these NT and OP compounds [10].

Chromogenic sensors and probes are inexpensive, easy to use, allow for on-site detection, and need little to no instrumentation; they offer an enticing alternative to standard instrumental approaches to overcome these limitations [11,12,13]. Recently, robust catalytically efficient enzyme-mimetics, known as nanozymes [14,15,16,17], have been used as optical sensing platforms that enable the highly sensitive detection of analytes based on color changes that can be observed by the naked eye [18,19,20,21,22]. Nanozymes exhibit intrinsic catalytic activity that is comparable to or greater than that of native enzymes, with no drawbacks such as poor durability or performance in a variety of harsh environmental conditions. Nanozyme-based biosensors display several advantages, such as (a) rapid detection and better sensitivity, (b) amplified and easier signal readout and naked-eye detection, and (c) easier accessibility and uniform detection capability by multiple users. The application of nanozymes in biosensors is a promising opportunity for the development of inexpensive and effective point-of-care devices for rapid detection. Recently the nanozyme activity of several classes of nanomaterials such as metal oxide-based particles, gold-silver core–shell nanoparticles [23], metal-organic frameworks (MOFs) [24], 3D porous materials [25], carbon-based materials [25] and nanoparticle-MOF composites [26] have been used to develop biosensing platforms. Despite these systems offering excellent catalytic activity, they are restricted by complicated synthesis protocols for core–shell nanoparticles, aggregation issues in platforms based on nanoparticles, limited water solubility, and limited options for post-synthetic modifications for these systems.

Supramolecular self-assembly provides a powerful methodology for building complex and ordered structures out of relatively simple building blocks using noncovalent interactions that have adaptable, dynamic, and responsive features [27,28]. Supramolecular entities can be designed either by the self-assembly of a single component or the co-assembly of multiple components. Multicomponent self-assembly approaches enable a wider range of more complex structures to be generated with enhanced modularity and offer better spatiotemporal control of self-assembly [29,30]. Supramolecular multicomponent assemblies with metal as one of the components offer the advantages of traditional nanozymes; however, organic ligands lead to the generation of ordered structures with larger surface areas and greater biocompatibility. The adaptability [30] and biocompatibility [31] of these self-assembled nanozymes help to achieve synthetic biomimetic systems effectively. Furthermore, such supramolecular self-assembled nanozymes have several advantages, such as outstanding catalytic activity, great water solubility, ease of synthesis, and facile operability under ambient and mild conditions [32,33]. Herein, we report a 3D supramolecular nanozyme (henceforth known as SupraZyme) system based on multicomponent self-assembled bilayer vesicles for the sensing of neurotransmitters and organophosphorus compounds. The designed system consists of Cu^2+^ coordinated to a tridentate ligand, iminodiacetic acid (IDA), and a synthesized ligand **L1** binding to two coordination sites of Cu^2+^. The vacant coordination site occupied by water molecules imparts multi-enzymatic activity to the assembled system. The peroxidase activity of the SupraZyme was utilized for the detection of EP and NE. The oxidase-like activity of SupraZyme was readily inhibited by thiocholine (TCh) and generated through the AChE-catalyzed hydrolysis of acetylthiocholine (ATCh). This interesting feature of SupraZyme was utilized in the sensitive detection of cholinesterase and OP chemicals. Overall, the supramolecular self-assembled nanozyme introduces a promising sensing platform for molecules of biological importance.

## 2. Experimental Section

### 2.1. Materials

Acetylcholine esterase (AChE, 1000 U/mg), bovine serum albumin, β-lactoglobulin, tyrosinol hydrochloride, lysozyme, acetylthiocholine chloride, and D,L-Norepinephrine (NE) hydrochloride were purchased from Sigma-Aldrich. Epinephrine (EP) and Hydrogen peroxide (H_2_O_2_) were procured from TCI chemicals. Other chemicals such as tyrosine, guaiacol, urea, sodium tripolyphosphate, potassium dihydrogen phosphate, disodium hydrogen phosphate, ammonium bicarbonate, ammonium sulfate, 4-amino antipyrine (4-AP), 2,4-dichlorophenol (2,4-DP), heptanoic acid, histamine dihydrochloride, iminodiacetic acid (IDA), copper nitrate (Cu(NO_3_)_2_·3H_2_O), dicyclohexylcarbodiimide (DCC), N-hydroxysuccinimide (NHS), dimethylformamide (DMF), dichloromethane (DCM), tetrahydrofuran (THF), sodium bicarbonate (NaHCO_3_), and methanol were obtained from SRL chemicals, India. The chemicals obtained were used without further purification. All the experiments were performed using Milli-Q water.

### 2.2. Fabrication of SupraZyme

A stock solution of IDA (10 mM) and copper nitrate (10 mM) was prepared in Milli-Q water. Ligand **L1** was synthesized as per the previous literature reports with some slight modifications (Scheme S1 in Supplementary Materials). The characterization of **L1** was performed using ^1^H-NMR and HR-MS (Figure S1). The stock solution of ligand **L1** (10 mM) was prepared in methanol.

A copper solution (10 μL, 10 mM), IDA (10 μL, 10 mM), and **L1** (20 μL, 10 mM) were added to 960 µL of water, mixed well, and left undisturbed for 1 h. The as-prepared SupraZyme was used for further experiments.

### 2.3. Sensing of Acetylcholine Esterase and Neurotransmitter Surrogate Acetylthiocholine

To determine the detection limit of AChE, the stock solution (1 mg/mL) and working solution (10 µg/mL) of AChE were prepared using 50 mM sodium phosphate buffer (pH 7.4). The AChE detection assay was established by pipetting 60 µL of a 5 mM phosphate buffer (pH 7.4). A total of 10 µL ATCh (20 mM) and varying concentrations of 10 µL of AChE were used. The mixture solutions were incubated in a 96-well plate at 37 °C for 20 min. Later, 100 µL of the SupraZyme was added and incubated for 30 min at room temperature (RT). Finally, 2,4-DP (1 mM) and 4-AP (1 mM) were added, and the absorption spectra of the resultant solutions were recorded after 30 min of incubation.

For the detection of ATCh, 10 µL of AChE (0.2 µg/mL) was added to various concentrations of ATCh (0 to 100 µM) and diluted with 60 µL of a 5 mM phosphate buffer (pH 7.4) in a 96-well plate. The mixture was incubated for 20 min at 37 °C, followed by the addition of 100 µL of the SupraZyme. After 30 min of incubation at RT, 2,4−DP (1 mM) and 4−AP (1 mM) were added, and the absorption spectra for the resulting solutions were read after 30 min.

### 2.4. Sensing of Neurotransmitter Epinephrine and Norepinephrine

Epinephrine (EP) stock solution was prepared in 12 mM HCl. EP solutions of different concentrations were freshly prepared by diluting the stock solutions in water at RT. Then, 10 μL of the EP solution at the corresponding concentration was added to 100 μL of the SupraZyme along with 80 μL of 100 mM HEPES buffer (pH 7.4). Thereafter, 10 μL of H_2_O_2_ (20 mM) was added, and the absorbance at 475 nm was recorded after 15 min of incubation at RT. The corresponding UV–vis spectra were recorded from 400 to 600 nm.

Norepinephrine (NE) stock solution was prepared in Milli-Q water and then diluted to varying concentrations in the water. In a 96-well plate, 10 μL from NE stocks of various concentrations and 100 μL of SupraZyme were added to a 100 mM HEPES buffer (pH 7.4) followed by the addition of 10 μL of H_2_O_2_ (20 mM) and, after 30 min of incubation at RT, the absorbance endpoint was recorded at 483 nm.

### 2.5. Sensing of Organophosphorus Pesticides

Various concentrations of POM (0 to 100 ppb) and MAP (0 to 500 ppb) were added to 10 µL AChE (0.2 µg/mL) in a 96-well plate and diluted with 50 µL of a 5 mM phosphate buffer (pH 7.4) and were incubated for 30 min at 37 °C. Further, ATCh (10 µL, 20 mM) was added and incubated for another 20 min at 37 °C. Later, 100 µL of as prepared SupraZyme assembly was introduced into the system, and following 30 min of incubation at 25 °C, 2,4-DP (10 µL, 20 mM) and 4-AP (10 µL, 20 mM) were added. After 20 min, the absorbance spectra were recorded from 400 to 650 nm, and the absorbance of the endpoint was recorded at 510 nm, corresponding to the absorbance of the quinone-imine product.

### 2.6. Sensing of Organophosphorus Pesticides in Spiked Samples

Urban tap water was collected and used as such without any purification step. The apple juice was prepared from an apple procured from a local shop and diluted to 10% (*v*/*v*) using MilliQ water and used as it was for further studies. Different concentrations of OP pesticide POM were spiked into the tap water and juice separately. Then, these spiked samples were utilized to detect POM according to the same steps mentioned above. The final concentration range screened for POM detection ranged from 0 to 100 ppb.

## 3. Results and Discussion

### 3.1. SupraZyme Preparation and Characterization

**Figure 1:**
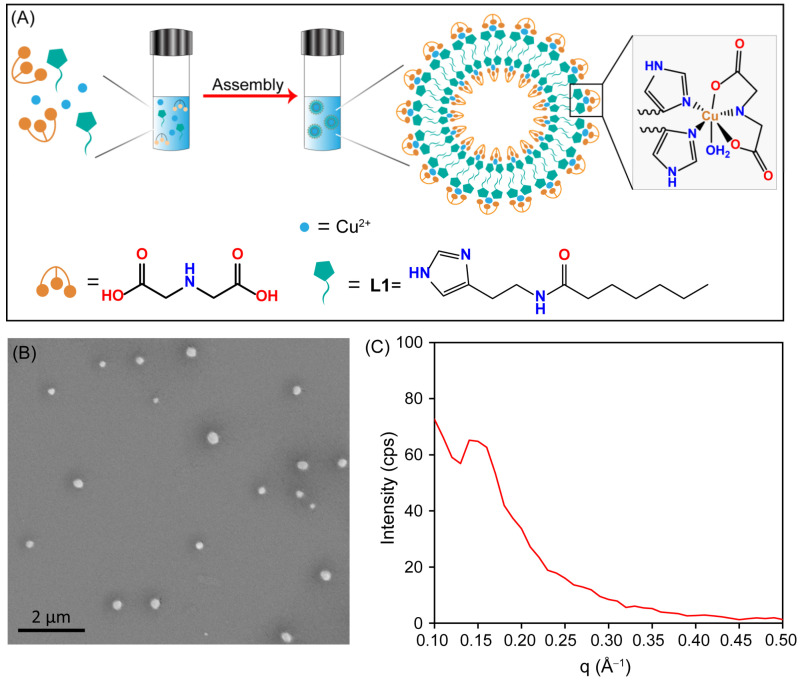
Fabrication of SupraZyme and its characterizations. (**A**) A schematic of assembly formation with Cu(NO_3_)_2_, IDA, and ligand **L1**. (**B**) SEM image of the SupraZyme showing vesicular structures, (**C**) SAXS spectrum of the SupraZyme showing a peak corresponding to the q value of 0.15 Å^−1^. Experimental conditions: [Cu^2+^] = 100 μM, [**L1**] = 200 μM, and [IDA] = 100 μM.

We have previously reported a metal coordination-directed dissipative supramolecular self-assembled system with Cu^2+^ as a catalytically active center [32]. The assembled system consists of a multivalent chelating ligand iminodiacetic acid (IDA), which coordinates to three coordination sites of the Cu^2+^ metal center and two ligand **L1** occupying two coordination sites through the imidazole headgroup. The synthesis scheme of Ligand **L1** (Scheme S1) and the ^1^H-NMR and HRMS characterization (Figure S1) are detailed in the Supplementary Materials. The self-assembled nanostructure obtained by incubating Cu^2+^, IDA, and ligand **L1** at a ratio of 1:1:2 in water generates bilayer vesicles (Figure 1A). The IDA ligand coordinates to the Cu^2+^ ion through one nitrogen and one oxygen in the vesicular SupraZyme in an equatorial manner, while the remaining oxygen coordinates through the axial position (Figure 1A). In the equatorial plane, the two imidazole moieties coordinate in a cis geometry. A labile water molecule coordinates the unoccupied axial binding site and gives the vesicular system its catalytic activity (Figure 1A). The scanning electron microscopy (SEM) images reveal a spherical morphology of size ~200 nm (Figure 1B). We used nanoparticle tracking analysis (NTA) to assess the particle size distribution and concentration of the vesicles. The diameter of the self-assembled SupraZyme was measured to be 150–200 nm, with a concentration of 2.06 × 10^8^ particles/mL (Figure S2). Further, the bilayer structure of the metallo-supramolecular assembly was validated through small-angle X-ray scattering (SAXS) analysis (Figure 1C). The *q* value of 0.15 Å^−1^ corresponds to a bilayer thickness (*d*) of 4.2 nm, which is calculated using Bragg’s equation of $d=\frac{2\pi }{q}$. The amide groups of the two **L1** molecules with cis-orientation in the assembled structure impart intermolecular hydrogen bonding, which strengthens the hydrophobic interaction between the alkyl chains and leads to a stable bilayer structure in the water. Altogether, the cis-geometry of the **L1** ligands coupled with the multivalent binding of IDA leads to an effective van der Waals interaction between the hydrophobic tail of **L1**, facilitated by the intermolecular H-bonding that results in the stable bilayer vesicular structures containing catalytically active Cu^2+^ centers.

### 3.2. Inhibition of Oxidase-like Nanozyme Activity by Thiol Groups

**Figure 2:**
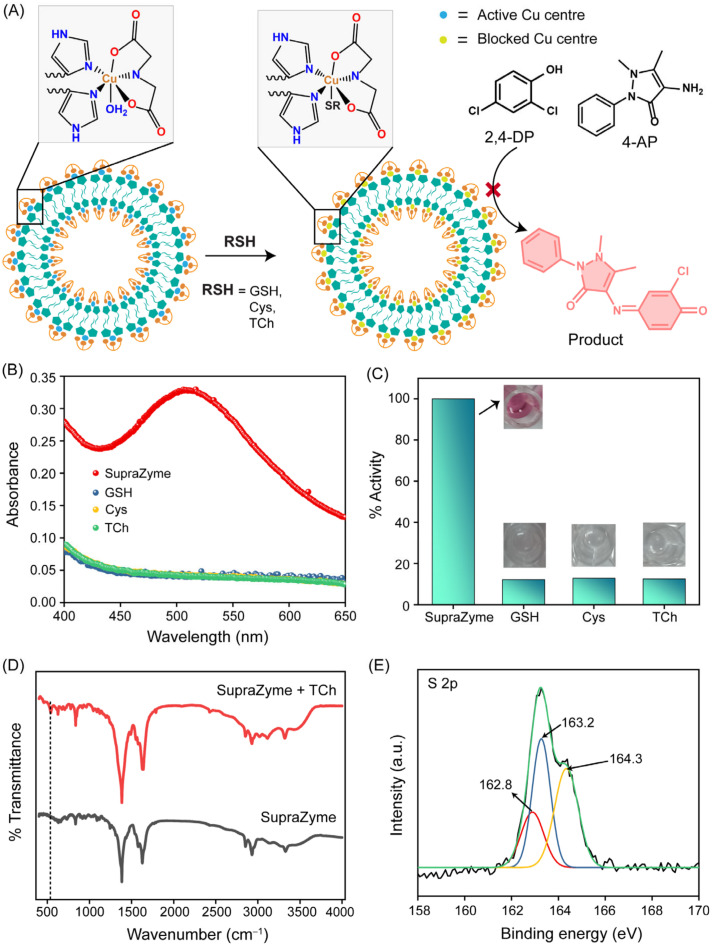
Catalytic activity blocking of SupraZyme. (**A**) Schematic for blocking oxidase activity of SupraZyme in the presence of thiols (RSH), where the blue Cu centers in the left panel imply a catalytically active center and in the right yellow Cu centers imply coordinatively saturated, catalytically inactive Cu centers failing the conversion of 2,4-DP and 4-AP to the product, (**B**) Absorbance spectra of the reaction of substrates 2,4-DP and 4-AP in the presence of SupraZyme and different thiols GSH, Cys and TCh (200 µM) and (**C**) Percentage of catalytic activity for the catalysis of imine formation in the presence of GSH, Cys, and TCh (200 µM); (**D**) FT-IR of SupraZyme in presence and absence of TCh; (**E**) XPS spectrum of S 2p in TCh blocked SupraZyme.

The Cu(II)-bearing SupraZyme exhibits oxidase-mimetic nanozyme activity that is similar to naturally occurring laccase enzymes [32]. The native laccase enzyme consists of four copper centers in the active site that catalyze the oxidation of a wide range of phenolic compounds, enabling environmental remediation. Our self-assembled material exhibits similar laccase-like activity due to its active copper center and catalyzes the reaction between chromogenic substrates 2,4-DP and 4-AP to form a pink-colored quinone–imine complex with an absorbance maximum at 510 nm in the presence of molecular oxygen. Mechanistic insights [32] revealed that the water molecule that coordinated at the axial site on Cu^2+^ of SupraZyme was replaced by the phenolic substrate 2,4-DP. Eventually, this 2,4-DP becomes oxidized to quinone with a subsequent reduction in Cu(II) to Cu(I). Further, the Cu(I) is oxidized to Cu(II) by the reduction in molecular oxygen, completing the catalytic cycle. The binding of the phenolic substrate to the Cu(II) center is a key step that leads to the oxidation of the substrate. Therefore, the labile water molecule in the coordination site of the SupraZyme is crucial for oxidizing phenolic substrate 2,4-DP and 4-AP to the quinone–imine complex (Figure 2A). We hypothesized that blocking this coordination site by a non-labile molecule can inhibit the oxidase-like activity of the SupraZyme (Figure 2A). To turn off the catalytic activity of SupraZyme, we tested thiols as the irreversible inhibitor owing to the strong binding affinity of thiols towards copper [34]. The oxidase-like activity of SupraZyme was probed in the presence of thiol-containing biomolecules such as glutathione (GSH), cysteine (Cys), and thiocholine (TCh). The absorbance at 510 nm, corresponding to the quinone–imine complex, was drastically diminished when the SupraZyme was incubated with 200 µM of thiols as the coordination site occupied by water became irreversibly coordinated to the sulfide group of GSH, Cys, and TCh (Figure 2B). The results revealed that the oxidase-like catalytic activity of our SupraZyme was >90% inhibited in the presence of these thiols (Figure 2C).

The thiol-mediated inhibition of SupraZyme activity was further investigated using different analytical techniques to learn about the interactions. To confirm the formation of the Cu-S bond, we performed the FT-IR analysis of SupraZyme with and without TCh. The comparison of the spectra revealed the origin of a new peak at 543 cm^−1^ corresponding to the new Cu-S bond formed (Figure 2D). To obtain more insight into the coordination between Cu and S, we carried out X-ray photoelectron spectroscopy (XPS) which showed three major peaks positioned at 162.8, 163.2, and 164.3 eV in the spectrum of S 2p (Figure 2E). The S 2p peak at 162.8 eV was ascribed to S–Cu, which revealed the existence of coordination between Cu and S; the peak at 163.2 eV is due to free sulfur species and 164.3 eV due to disulfide bonds [35]. Therefore, the results validate the affinity of sulfur to the Cu(II) ion, which results in nanozyme activity inhibition.

### 3.3. Oxidase-like Nanozyme Activity to Generate Colorimetric Response for Biosensing

**Figure 3:**
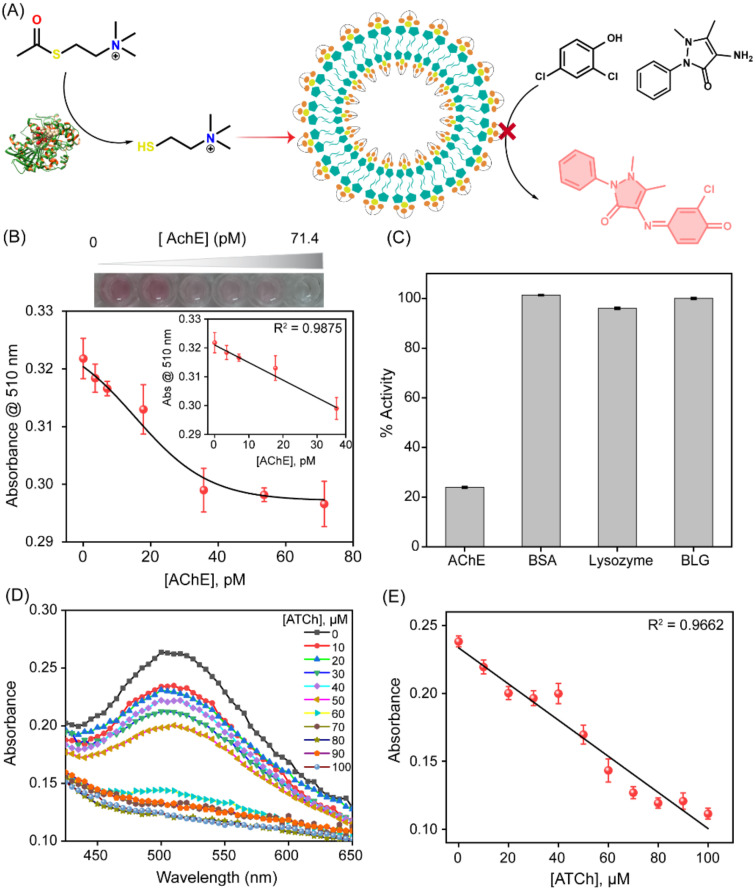
AChE and ATCh sensing. (**A**) Schematic of the activity inhibition of SupraZyme for the colorimetric detection of AChE, wherein ATCh generated by AChE inhibits the oxidase-like activity of SupraZyme. (**B**) Plot of the absorbance values at 510 nm as a function of the AChE concentrations (0 to 71.4 pM) at [ATCh] = 1 mM; the black line represents the dose–response curve fitting to guide the eye, inset shows the linear fitting of the data with a detection limit of 17.2 pM (**C**) The selectivity of SupraZyme activity inhibition, at a wavelength of 510 nm, by AChE and other proteins (concentration = 35 pM) in the presence of 1mM ATCh. (**D**) UV-Vis spectra for oxidase activity of SupraZyme at varying concentrations of ATCh (0 to 100 µM) at [AChE] = 35 pM, and (**E**) Plot of absorbance values at 510 nm as a function of the varying concentration of ATCh (0 to 100 µM), the black line represents the linear fitting of the data giving a detection limit of 9 µM.

The thiol-mediated inhibition of nanozyme activity motivated us to explore the detection of the enzyme acetylcholine esterase by the SupraZyme system. AChE acts as a catalyst for the hydrolysis of acetylthiocholine (ATCh) to TCh. The generated TCh can bind to the vacant binding site on the Cu^2+^ center and thus inhibit the catalytic activity of the SupraZyme (Figure 3A). This principle was used to detect the levels of AChE in the solution. It was observed that the SupraZyme activity gradually decreased with the increase in AChE concentration (Figure S3). The limit of detection (LOD) of AChE was found to be 17.2 pM (or 4.8 mU/mL) (Figure 3B), which is a biologically relevant concentration. The limit of detection was calculated using the formula of 3σ/S, where σ is the standard deviation of three blank samples and S is the slope of the obtained linear fitted graph. We probed the robustness of the SupraZyme system through the inter and intra-batch variability. Two different sets of SupraZyme were tested for their activity inhibition against AChE (35 pM) and ATCh (1 mM), as shown in Figure S4. The results indicate that the % of activity was reduced to less than 20% for all the intra-batch samples (A, B, and C in Figure S4). Likewise, two different batches showed similar inhibition of SupraZyme activity (Set 1 and 2 in Figure S4). Significantly, the sensor features a good selectivity for the AChE protein, as evident from the unchanged SupraZyme activity by other proteins, such as bovine serum albumin (BSA), Lysozyme, and β-lactoglobulin (BLG), in the presence of ATCh (Figure 3C). However, the oxidase-like activity of SupraZyme is inhibited down to 20% by the same concentration of AChE at the same experimental condition.

Acetylthiocholine detection is used as a surrogate for the detection of the neurotransmitter acetylcholine. We utilized the inhibition of SupraZyme catalytic activity to detect acetylthiocholine levels. The increasing concentration of ATCh leads to the generation of higher concentrations of thiocholine, which turns “off” the oxidase-like catalytic activity of the SupraZyme. Hence, the conversion of 2,4-DP and 4-AP into their corresponding imine product is reduced, leading to the dampening of absorbance at 510 nm corresponding to the absorbance maximum of the product (Figure 3D). The absorbance values at 510 nm were plotted against the varying concentrations of ATCh (0 to 100 µM) (Figure 3E)). The UV-Vis spectrometric response follows a linear trend in this concentration range. The full assay takes nearly 70 min to complete, including the incubation steps, and the LOD was determined to be 9 µM. The performance of our SupraZyme strategy for the sensing of AChE and ATCh was comparable and even superior to some of the reported assays (Table S1). Therefore, the nanozyme platform provides a sensitive colorimetric biosensing tool for cholinergic targets.

### 3.4. Detection of Epinephrine and Norepinephrine

**Figure 4:**
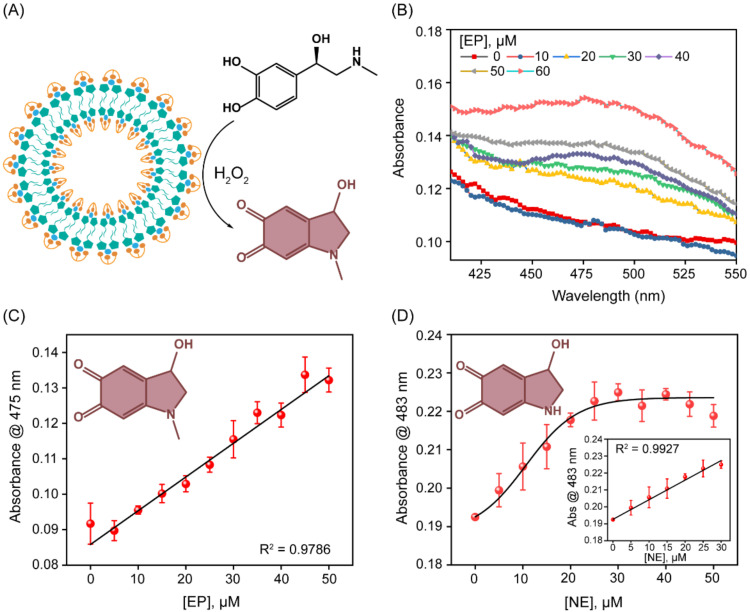
SupraZyme catalyzed oxidation of EP and NE. (**A**) Graphical representation of EP oxidation to adrenochrome using SupraZyme, (**B**) UV-Vis absorbance spectra obtained by oxidation of EP (0 to 60 µM) in the presence of SupraZyme at H_2_O_2_ = 1 mM (**C**) Absorbance at 475 nm as a function of EP concentration varying from 0 to 50 μM at H_2_O_2_ = 1 mM. The black line represents a linear fitting of the data giving a detection limit of 6.3 µM while the brown colored product is the oxidized product adrenochrome, (**D**) Plot of absorbance at 483 nm as a function of NE concentrations varying from 0 to 50 μM at 1 mM H_2_O_2_ where the oxidized product noradrenochrome is presented in brown color. The black line represents the dose-responsive curve fitting for guiding the eye and the inset shows the linear fitting of the data and corresponding limit of detection for NE is 1.8 µM.

The peroxidase-like catalytic activity of the SupraZyme was first tested for EP detection. EP is an important neurotransmitter that is responsible for the fight or flight response of our body and the dysregulation of which is associated with several disease conditions, including cardiac arrhythmia, psychosocial stress, depressive disorders, and neurodegenerative diseases such as Parkinson’s and Alzheimer’s [36,37]. We investigated the SupraZyme-catalyzed oxidation of EP to adrenochrome by H_2_O_2_ (Figure 4A). The extent of oxidation was quantified using the absorption maximum corresponding to the adrenochrome product that was generated (λ_max_: 475 nm). As the concentration of EP increases, the catalytic oxidation of EP to adrenochrome intensifies (Figure 4B). The absorbance values at 475 nm after 15 min were plotted against different concentrations of EP, and the absorbance values showed a linear increase in the concentration range from 0 to 50 µM (Figure 4C). The lowest concentration of EP that could be detected using the catalytic assay was 6.3 μM as calculated from the 3σ/slope. The overall assay time for EP oxidation was 30 min, while the detection time was 15 min. To investigate the inter and intra-batch variation in SupraZyme when detecting EP, two different batches of SupraZyme were used (Set 1 and Set 2), and the corresponding linear dependence curve with an increasing concentration of EP was used as depicted in Figure S5. The LOD calculated from the two sets were 6.3 μM and 6.9 μM, respectively, confirming the robustness of the biosensor utilizing the peroxidase-like activity. The selectivity for EP detection was probed through the oxidation of other small molecules bearing dihydroxyl and amine groups, such as tyrosine, tyrosinol, hydroquinone, and guaiacol using the peroxidase-like activity of SupraZyme. The UV-Vis traces for all the test molecules showed considerable absorbance at 483 nm for EP only (Figure S6), demonstrating the selectivity of the biosensor for EP detection over other similar small molecules.

To probe the versatility of the sensor in detecting catecholamine containing neurotransmitters, we investigated another important catecholamine norepinephrine (NE). NE is shown to have a connection with major depressive disorders and psychiatric conditions such as schizophrenia [37,38]. It was observed that the SupraZyme effectively oxidized NE to noradrenochrome, which showed an absorption maximum at 296 and 483 nm (Figure S7). The absorbance maxima at 483 nm were plotted against the increasing concentration of NE (Figure S8). The plot followed a linear trend and the LOD corresponding to NE was found to be 1.8 µM (Figure 4D). The detection of NE takes ~30 min while the overall assay requires around 45 min to complete. The analytical performance of SupraZyme compared to other reported assays (Table S2) indicates the suitability of SupraZyme for the determination of catecholamine neurotransmitters EP and NE in point-of-care detection.

### 3.5. Detection of Organophosphorus (OP) Pesticides

**Figure 5:**
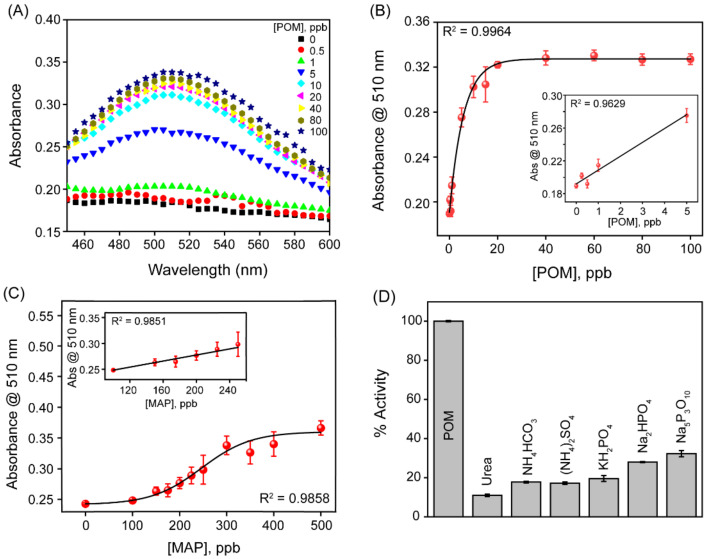
Detection of organophosphorus pesticides. (**A**) UV-Vis absorbance spectra of varying POM concentrations (from 0 to 100 ppb). (**B**) Plot of absorbance values at 510 nm as a function of POM concentration, the black line indicates the dose–response curve fitting while linear fitting in the inset. (**C**) Plot of absorbance values at 510 nm as a function of MAP concentration, the black line indicates the dose–response curve fitting to guide the eye and the inset shows the linear fitting of data, (**D**) Selectivity of POM detection in comparison to other commonly used fertilizers and phosphorus containing small molecules at 100 ppb.

The detection mechanism of OP compounds is based on the inhibition of AChE activity by the SupraZyme, preventing the production of TCh, which reinstates the oxidase-like activity of SupraZyme. In the absence of OP compounds such as paraoxon-methyl (POM) and methamidophos (MAP), AChE catalyzes the hydrolysis of ATCh to generate TCh. This generated TCh binds to the unoccupied coordination site of Cu^2+^ through its thiol group and inhibits oxidase mimetic activity. However, in the presence of increasing concentrations of POM, the amount of TCh generated reduces successively, and the oxidase mimetic catalytic activity shows an increasing trend, i.e., the absorbance maximum at 510 nm increases with increasing concentrations of OP compounds (Figure 5A). The absorbance at 510 nm was plotted at varying concentrations of POM. The dose–response curve fitting for POM is shown in Figure 5B, while the inset shows the dynamic range. The detection limit of POM was determined to be 0.48 ppb following the linear fitting of the dynamic range for POM. To assess the versatility of SupraZyme for detecting OP toxins, we chose another broad-spectrum OP insecticide, methamidophos, which is sold under several trade names (Metafort, Metaphos, Metamidofos Estrella, Methafos, and Monitor). The absorption spectra for increasing concentrations of MAP shows an upward trend (Figure S9), and the detection limit for MAP was found to be 15.8 ppb from the linear fitting of the dynamic range (Figure 5C). Commonly used fertilizers in agriculture such as urea, ammonium bicarbonate (NH_4_HCO_3_), ammonium sulfate ((NH_4_)_2_SO_4_), and phosphorus-containing small molecules such as KH_2_PO_4_, Na_2_HPO_4,_ and sodium triphosphate (Na_5_P_3_O_10_) were used to test the selectivity of the biosensor as shown in Figure 5D. None of these compounds were able to restore the catalytic activity of SupraZyme effectively (<35%) in comparison to the 100% catalytic activity restored by the OP pesticide, indicating the better selectivity of the SupraZyme to detect OPs. The detection time after the addition of the substrates was around 20 min while the whole assay time for the pesticide detection was nearly 100 min. Although the detection time for the pesticides is apparently high from the perspective of point-of-care detection, the assay duration could be significantly reduced by optimizing the substrate concentrations (2,4-DP and 4-AP) as well as increasing the concentration of SupraZyme.

The performance of SupraZyme compared with that of other reported OP pesticide biosensors is summarized in Table 1 [39,40,41,42,43,44,45,46,47,48]. It is evident that the SupraZyme performs better than other colorimetric methods and is comparable to electrochemical methods.

**Figure 6:**
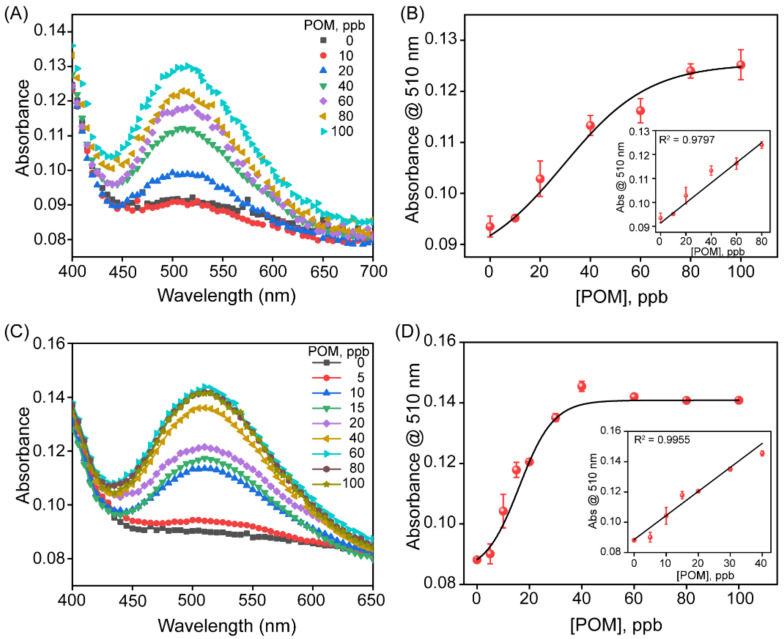
Detection of paraoxon methyl in complex matrices. (**A**) UV-Vis absorbance spectra of varying POM concentrations (from 0 to 100 ppb) spiked in tap water. (**B**) Plot of absorbance values at 510 nm as a function of POM concentration spiked in tap water, the black line indicates the dose–response curve fitting to guide the eye while linear fitting in the inset gives the LOD of 14.5 ppb. (**C**) UV-Vis absorbance spectra of varying POM concentrations (from 0 to 100 ppb) spiked in apple juice. (**D**) Plot of absorbance at 510 nm with increasing POM concentration spiked in apple juice, where the black line indicates the dose–response curve fitting to guide the eye. The inset shows the linear fitting of data with a detection limit of 0.77 ppb.

To investigate the practical utility of the sensor, we probed the detection efficiency of OP pesticides in urban tap water and an apple juice extract spiked with varying concentrations of POM (0 to 100 ppb). The increasing concentration of POM spiked in tap water showed an increase in the absorbance (Figure 6A). A good linear relationship was observed between the increase in absorbance at 510 nm with the increasing POM concentration in spiked tap water samples in a range from 0 to 100 ppb (Figure 6B). The LOD was calculated to be 14.5 ppb using the same formula (3σ/S). In the case of POM spiked in the apple juice extract, the absorbance spectra demonstrated an increase in absorbance with increasing concentrations of spiked POM, as shown in Figure 6C. The plot of absorbance at 510 nm versus the POM concentration showed a linear trend in the concentration range from 0 to 40 ppb with a correlation coefficient of 0.9955 (Figure 6D). The detection limit was calculated to be 0.77 ppb.

The LOD for both the OP pesticides detected were lower than 20 ppb, even in real-world matrices, which is below the maximum residue limit (MRL) for fruits and vegetables adopted by the European Commission, United States, and Canada [49]. Overall, the designed assay is easy to use, sensitive, and highly specific for detecting OP chemicals. As a result, the suggested sensor is highly sensitive and precise and can detect OP pesticides on-site and reliably. Since the class of organophosphorus pesticides can inhibit the activity of the AChE enzyme, the advantage of this strategy is limited to the detection of OP pesticides in a sample, while it cannot specifically distinguish between the various OP pesticides such as POM and MAP (used in this study). Other spectrometric and chromatographic methods would be required to identify if a particular OP pesticide was present. However, given that the aim of this study was to develop a point-of-care (PoC) detection strategy for OP pesticides, the delineation of OP pesticides in a mixed sample would not be necessary. The current method can serve as a good initial screening test and a complementary method for the PoC detection of OP pesticides in agricultural and food samples.

## 4. Conclusions

In this study, a sensitive biosensor was constructed based on supramolecular self-assembly that exhibited excellent multi-enzymatic activity. The peroxidase-like activity was exploited for the sensitive detection of the catecholamine-bearing neurotransmitter EP and NE with a lower detection limit of 6.3 µM and 1.8 µM, respectively, while the oxidase-like activity of the SupraZyme was employed for AChE and ATCh sensing. AChE-inhibition methods have been widely applied for the detection of residual OP pesticides in food and the environment and further the detection of OP pesticides such as POM and MAP, which has a crucial bearing on public safety and environmental protection, to very low detection limits of 0.48 ppb and 15.8 ppb, respectively. The detection limit of POM in spiked samples of tap water and apple juice extract was 14.5 ppb and 0.77 ppb, respectively, which is lower than the MRL of pesticides by regulatory agencies. The escalation of pesticide poisoning and environmental hazards has led to the growing demand for more innovative technologies and the commercial implementation of point-of-care biosensors for the detection of pesticides which can reduce both the time and costs involved. Altogether, this work demonstrates a 3D self-assembled supramolecular nanozyme as an effective strategy for point-of-care biosensing and sustainable environmental protection.

## Figures and Tables

**Table 1:** Comparison of the performance of biosensors for the detection of pesticides.

Method	Material	Pesticide Detected	Detection Limit	Ref.
Electrochemical methods	AChE/SWCNT-Co phthalocyanine/GCE	Paraoxon	2 ppb	[39]
	PPy-AChE-Geltn-Glut/Pt	Paraoxon	1.1 ppb	[40]
	manganese dioxide nanosheets (MnNS)	Paraoxon	0.025 ppb	[41]
	CeO_2_ nanozyme	Paraoxon	14.8 ppb	[42]
Colorimetric methods	RB-AuNPs	Ethoprophos	89 ppb	[43]
	Cysteamine capped gold nanoparticles (C-AuNPs)	parathion ethyl	5.8 ppb	[44]
	Fe_3_O_4_ magnetic nanoparticle	Paraoxon	2.47 ppb	[45]
	Nanoceria	Paraoxon	103.7 ppb	[46]
	AuNPs	Dimethoate	4.7 ppb	[47]
	Co_3_O_4_ Nanoplates	Glyphosate	175 ppb	[48]
	SupraZyme	Paraoxon	0.48 ppb	This work
		Methamidophos	15.8 ppb	

## Data Availability

The data that support the findings of this study are available from the corresponding author upon reasonable request.

## Supplementary Materials

The following supporting information can be downloaded at: https://www.mdpi.com/article/10.3390/bios13020277/s1, Scheme S1: Experimental Scheme for the synthesis of ligand L1; Figure S1: ^1^H NMR spectrum of synthesized ligand **L1**; Figure S2: NTA trace of the SupraZyme system; Figure S3: Absorbance spectra for oxidase activity of assembly at varying concentrations of AChE (0 to 71.4 pM) at [ATCh] = 1 mM; Figure S4: Percentage Activity of SupraZyme before and after addition of AChE (35 pM) and ATCh (1 mM) where Set 1 and Set 2 indicate inter-batch variation while A, B, C represents the intra-batch variation; Figure S5: Plot of absorbance @ 475 nm with varying concentrations of EP (0 to 50 µM) at [H_2_O_2_] = 1mM where Set 1 and Set 2 indicate inter-batch variation and error bars represent intra-batch variation; Figure S6: Selectivity of EP detection in comparison to other hydroxyl and amine-containing small molecules. UV-Vis absorbance spectra of oxidation of EP in comparison to other compounds at 100 µM concentration by peroxidase-like activity of SupraZyme where H_2_O_2_ = 1 mM; Figure S7: UV-Vis spectrum for noradrenochrome with λmax 296 and 483 nm; Figure S8: UV-Vis absorbance spectra obtained by oxidation of NE (0 to 50 µM) in the presence of SupraZyme at H_2_O_2_ = 1 mM; Figure S9: UV-Vis absorbance spectra of varying MAP concentrations (0 to 500 ppb); Table S1: Performance of Biosensors for detection of AChE and ATCh; Table S2: Performance of biosensors for detection of Neurotransmitters. Supplementary references [21,50,51,52,53,54,55,56,57,58,59,60,61,62,63,64,65,66,67].
