# Supplementary material for: Metal Coordination-Driven Supramolecular Nanozyme as an Effective Colorimetric Biosensor for Neurotransmitters and Organophosphorus Pesticides
Source: Biosensors (Basel). 2023 Feb 15;13(2):277. doi: 10.3390/bios13020277 (PMC9954067; doi:10.3390/bios13020277)
Supplement: Supplementary file 1 [file biosensors-13-00277-s001.zip › biosensors-2199013-supplementary.pdf]

biosensors

MDPI

Supplementary Material

Metal Coordination-Driven Supramolecular Nanozyme as an Effective Colorimetric Biosensor for Neurotransmitters and Organophosphorus Pesticides

Preeti Bhatt, Manju Solra, Smarak Islam Chaudhury and Subinoy Rana *

1. Instrumentation

^{1}H-NMR was recorded on a Bruker AV400 spectrometer operating at 400 MHz. The UV-Vis absorption spectra and kinetic analyses were acquired at room temperature using a Molecular Devices SpectraMax® M5e microplate reader. Field emission scanning electron microscope (FESEM) images were acquired using the Ultra55 FE-SEM Karl Zeiss EDS instrument with 5 kV emission voltage. X-ray photoelectron spectroscopy (XPS) was procured using K-Alpha X-ray Photoelectron Spectrometer, Thermo Scientific. Fourier-transform infrared spectroscopy (FT-IR) Spectra were recorded in a Shimadzu IR Affinity-1S instrument. Small angle X-ray Scattering (SAXS) was recorded on Small Angle X-ray Scattering (SAXS) Low Angle instrument from Nanostar.

2. Characterizations of SupraZyme

2.1. Field Emission Scanning Electron Microscopy (FESEM)

The SupraZyme self-assembly was freshly prepared and then 2 μL was drop-casted on a cleaned silicon wafer (multiple washes with IPA followed by acetone) and left for air-drying overnight. The sample was then put in a vacuum before recording the micrographs.

2.2. Small-angle X-ray Scattering (SAXS)

To prepare the powder sample, SupraZyme and SupraZyme incubated with thiol was lyophilized. The freeze-dried sample was then transferred to the sample holder and the data was measured using Nanostar Small Angle X-ray Scattering (SAXS) Low angle instrument.

2.3. Nanoparticle Tracking Analysis (NTA)

NTA determines the particle size distribution of samples in a liquid suspension, employing both Brownian motion and light scattering features. The prepared SupraZyme solution (1 mL) was injected to the sample chamber using 1 mL syringe and illuminated by the laser. The concentration and size of SupraZyme was analysed using NTA software.

3. Characterization of inhibition of oxidase-like activity of SupraZyme by thiols

3.1. X-ray photoelectron spectroscopy (XPS)

The prepared SupraZyme and SupraZyme incubated with thiol was lyophilized and sprinkled on a silicon wafer. The sample was kept in a vacuum till measurement.

3.2. Fourier-transform infrared spectroscopy (FTIR)

The prepared SupraZyme and SupraZyme incubated with thiol was lyophilized and the powdered sample was mixed with KBr to make the pellets and the spectra were recorded. Background measurements were made using a pellet of KBr only (without the sample).

4. Synthesis of Ligand L1

Synthesis of L1 was carried out following as per the previously reported protocol. For synthesis of Ligand, a solution of histamine (237.45 mg, 1.29 mmol) and sodium bicarbonate (144.48 mg, 1.72 mmol) in DMF/water (6 mL, 1:1 v/v) was kept at 0 °C. To this solution, a DMF solution of heptanoate-NHS ester (200 mg, 0.86 mmol) was added dropwise over a period of 10 minutes. The solution was stirred overnight under refluxing conditions. The product was extracted from the reaction mixture using chloroform. The organic layer was then dried over anhydrous Na_2SO_4, filtered, and concentrated using a rotatory evaporator. The product was recrystallized in DCM and n-hexane mixture leading to a solid white product. NMR and mass spectroscopy confirmed the formation of the product.

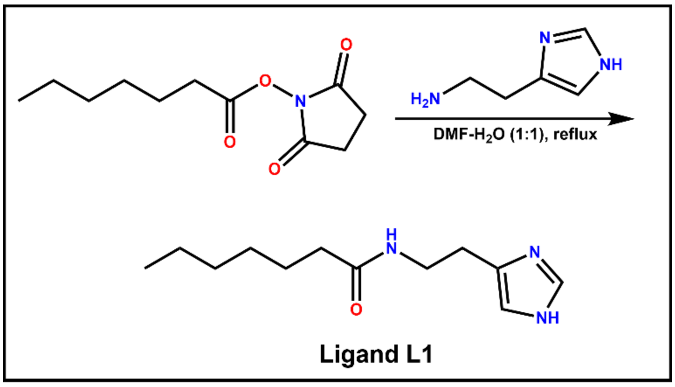

Scheme S1. Experimental Scheme for the synthesis of ligand **L1.**

5. Optimization of AChE and ATCh concentrations for sensing studies

Inhibition of SupraZyme activity by the produced thiocholine (TCh) is dependent on the concentration of both ATCh and AChE present in the solution. Hence, achieving the maximum inhibition of the SupraZyme activity is important to reduce the background to the minimum. We started the optimization with two objectives, (a) determining the lowest possible concentrations of ATCh and AChE for the assay, (b) ensuring faster kinetics of thiocholine generation from a point-of-care detection perspective. The LOD data for AChE (Figure 3B, Section 3.3) gave us a clue that the minimum concentration required to achieve maximum inhibition of SupraZyme activity is 35 pM. So, we fixed the AChE concentration to 35 pM, which corresponds to 10 µL of 0.2 µg/mL AChE, for the determination of the ATCh LOD as well as further studies of pesticide detection. Further, the final concentration of ATCh was chosen to be 1 mM, corresponding to 10 µL of 20 mM ATCh, for the AChE detection. A higher concentration of ATCh was chosen to ensure that the kinetics of ATCh hydrolysis is fast for the point-of-care detection.

6. Supplementary Figures

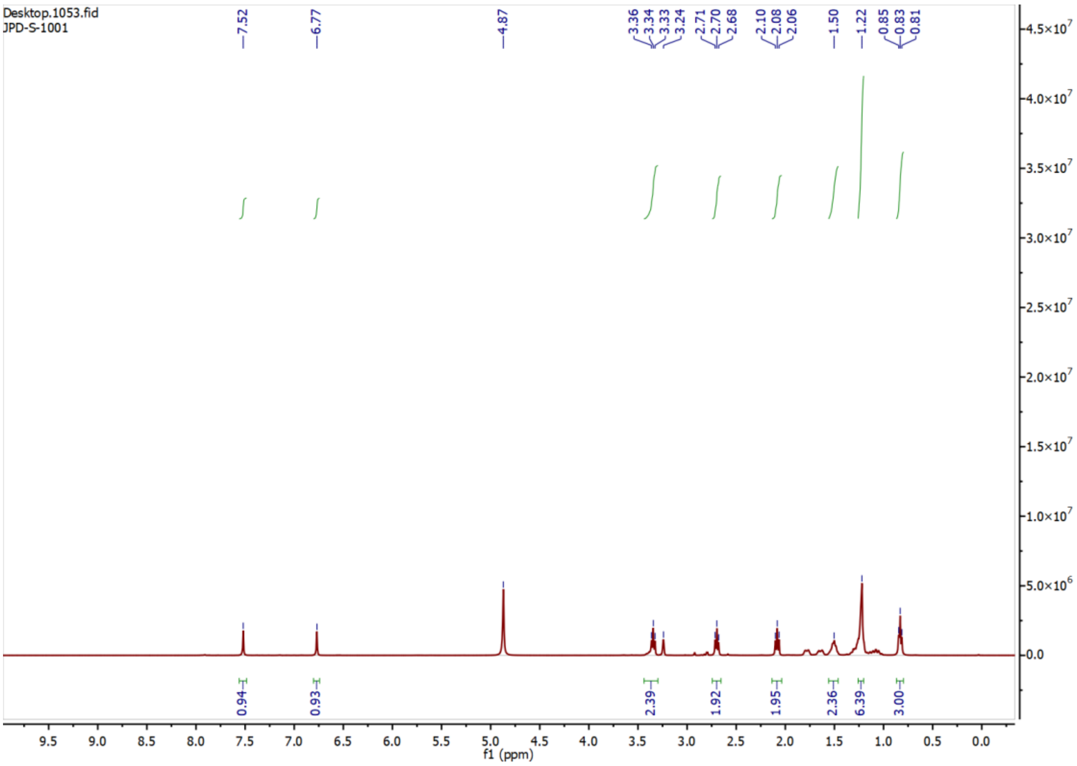

Figure S1. ^{1}H NMR spectrum of synthesized ligand **L1.**

Ligand L1: ^{1}H NMR (400 MHz, CD_3OD, δ (ppm)): 7.52 (s, 1H), 6.77 (s,1H), 3.34 (t, 2H), 2.70 (t, 2H), 2.08 (t,2H), 1.50 (m, 2H), 1.22 (m, 6H), 0.83 (t, 3H).

HRMS: Calculated mass of [**L1**] = 223.1685 m/z and Experimental mass [**L1**+H]$^+$ = 224.1763 m/z.

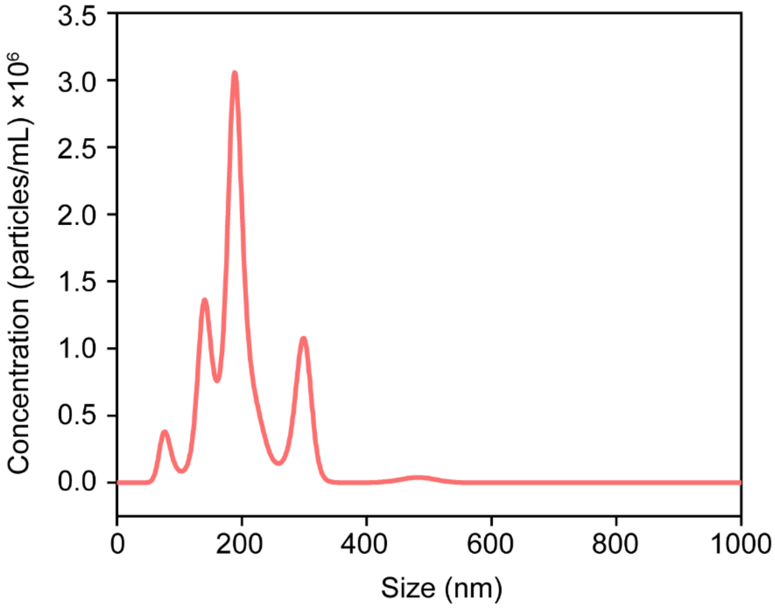

Figure S2. NTA trace of the SupraZyme system.

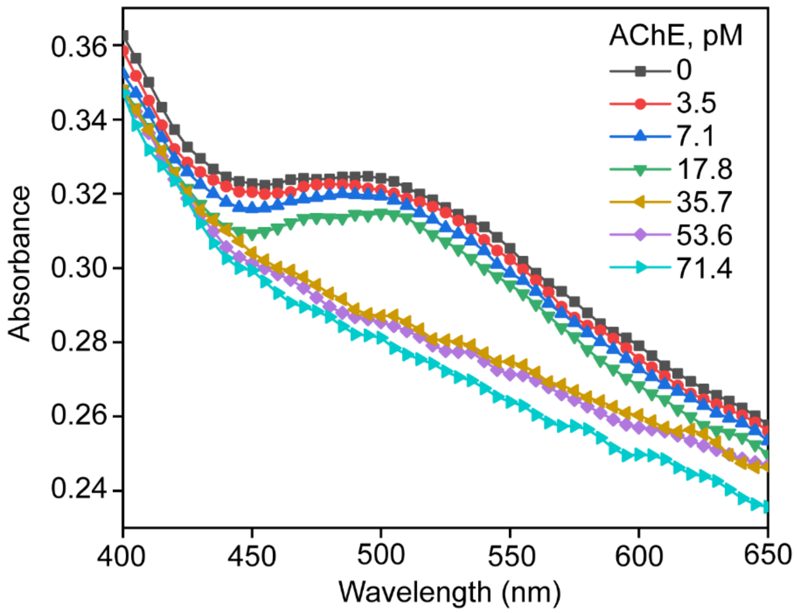

Figure S3. Absorbance spectra for oxidase activity of assembly at varying concentrations of AChE (0 to 71.4 pM) at [ATCh] = 1 mM.

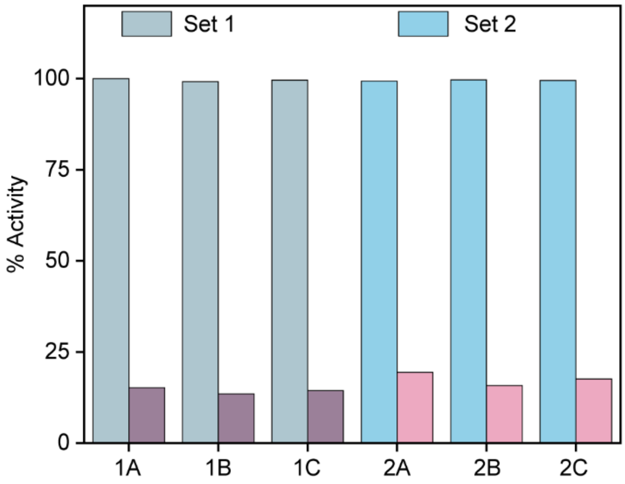

Figure S4. Percentage Activity of SupraZyme before and after addition of AChE (35 pM) and ATCh (1 mM) where Set 1 and Set 2 indicate inter-batch variation while A, B, C represents the intra-batch variation.

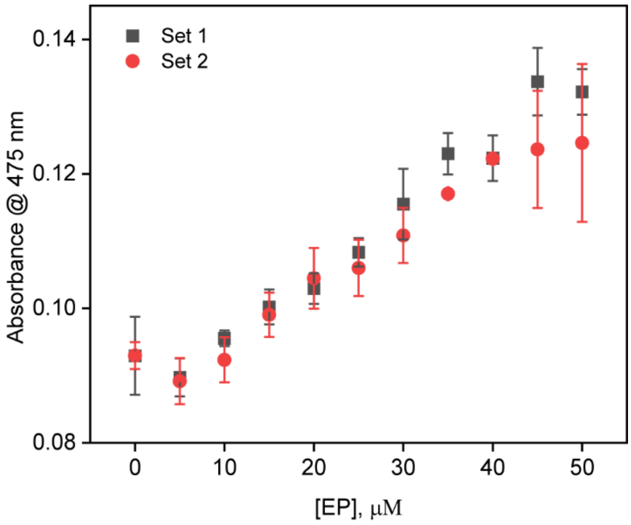

Figure S5. Plot of absorbance @ 475 nm with varying concentrations of EP (0 to 50 μM) at [H_2O_2] = 1mM where Set 1 and Set 2 indicate inter-batch variation and error bars represent intra-batch variation.

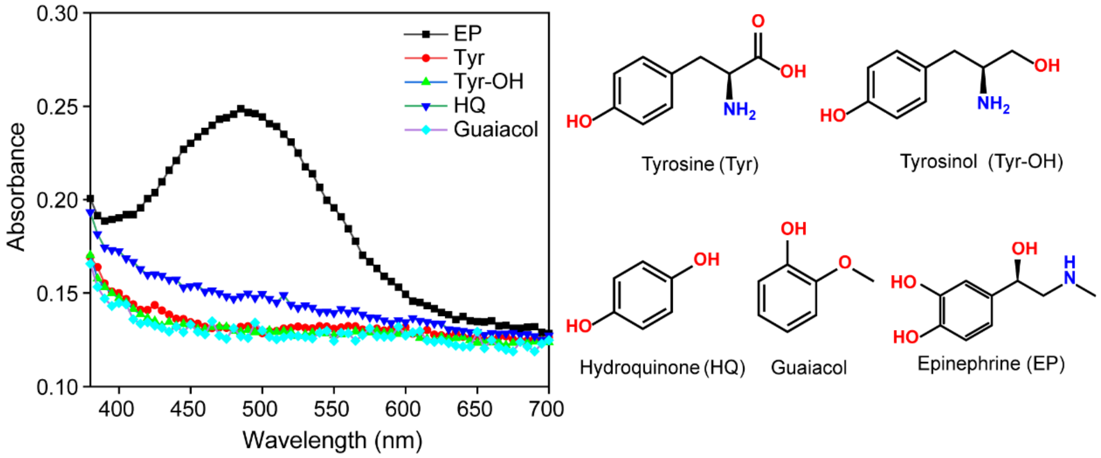

Figure S6. Selectivity of EP detection in comparison to other hydroxyl and amine-containing small molecules. UV-Vis absorbance spectra of oxidation of EP in comparison to other compounds at 100 μM concentration by peroxidase-like activity of SupraZyme where H_2O_2 = 1 mM.

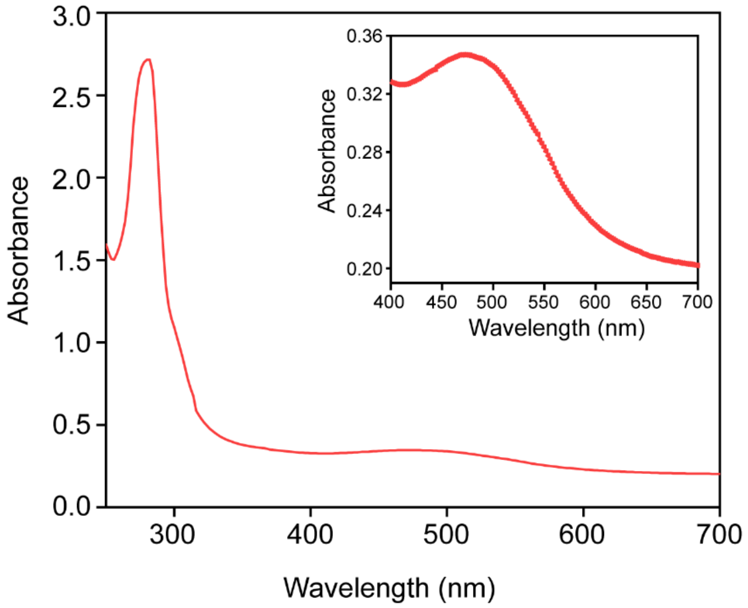

Figure S7. UV-Vis spectrum for noradrenochrome with λ_{max} 296 and 483 nm.

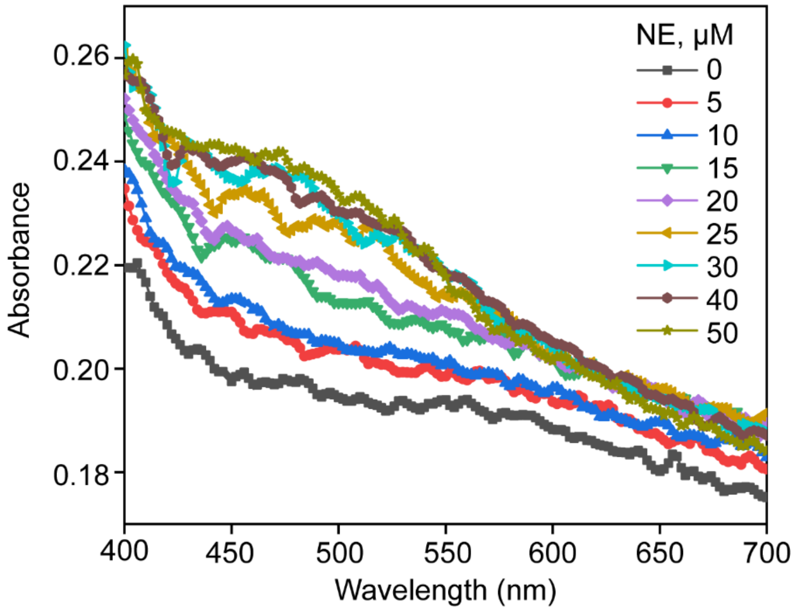

Figure S8. UV-Vis absorbance spectra obtained by oxidation of NE (0 to 50 µM) in the presence of SupraZyme at H_2O_2 = 1 mM.

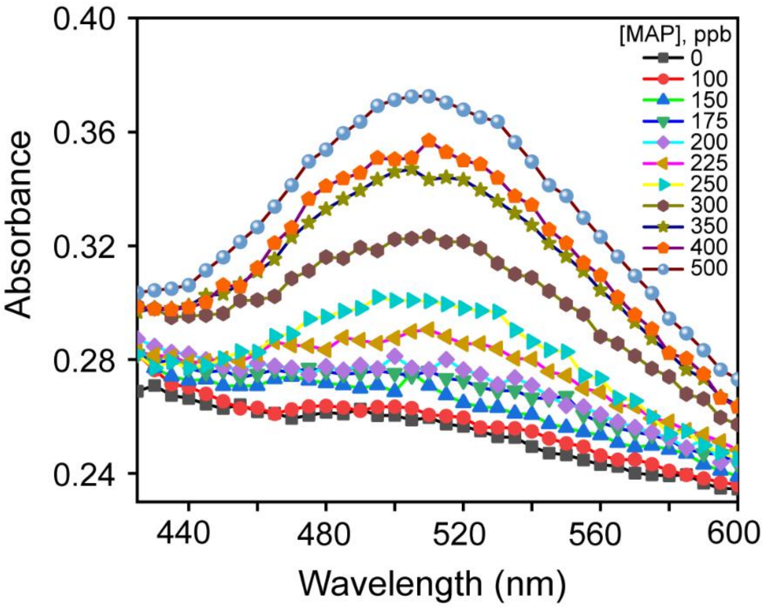

Figure S9. UV-Vis absorbance spectra of varying MAP concentrations (0 to 500 ppb).

Table S1. Performance of Biosensors for detection of AChE and ATCh.

Method	Target analyte	Detection limit	Reference
p-sulfonatocalix[6]arene (p-SC6A) capped AuNPs	AChE	0.16 mU/mL	[1]
γ-MnOOH nanozyme	AChE	0.1 mU/mL	[2]
Carbon quantum dots	AChE	4.25 mU/mL	[3]
Gold Nanoparticles	AChE	0.6 mU/mL	[4]
amine-terminated polydiacetylene vesicles	AChE	10.0 mU/mL	[5]
black phosphorus quantum dots (BP QDs)	AChE	0.17 mU/mL	[6]
Fe–N–C nanozyme	AChE	1.9 mU/mL	[7]
Peroxidase-like activity of acetyl-choline	ATCh	20.22 nM	[8]
multiwall carbon nanotube (MWNT)	ATCh	0.10 μM	[9]
electrochemical sensor	ATCh	25.28 μM	[10]
SupraZyme	AChE	4.8 mU/mL	This work
	ATCh	9 μM	

Table S2. Performance of biosensors for detection of Neurotransmitters.

Method	Target analyte	Detection limit	Reference
Rh-N/C-based biosensor	Epinephrine	0.57 μM	[11]
hexagonal prism Cu MOF	Epinephrine	1.1 μM	[12]
copper-cystine nanoleaves	Epinephrine	2.7 μM	[13]
Cu_2O nanospheres	Epinephrine	10 μM	[14]
CuO nanorods	Epinephrine	0.31 μM	[15]
Functionalized Gold Nanoparticles	Norepinephrine	0.07 μM	[16]
silver nanoparticles	Norepinephrine	5.59 μM	[17]
citrate-capped colloidal gold nano-particles (AuNPs)	Norepinephrine	42.2 μM	[18]

Polyacrylic Acid-Coated Nanoceria	Norepinephrine	0.126 μM	[19]
SupraZyme	Epinephrine	6.3 μM	This work
	Norepinephrine	1.8 μM	

Reference

1. Lv, J.; He, B.; Wang, N.; Li, M.; Lin, Y. A Gold Nanoparticle based Colorimetric and Fluorescent Dual-Channel Probe for Acetylcholinesterase Detection and Inhibitor Screening. *RSC Adv.* **2018**, *8*, 32893-32898.
2. Huang, L.; Sun, D.-W.; Pu, H.; Wei, Q.; Luo, L.; Wang, J. A Colorimetric Paper Sensor Based on the Domino Reaction of Acetylcholinesterase and Degradable γ-MnOOH Nanozyme for Sensitive Detection of Organophosphorus Pesticides. *Sens. Actuators B Chem.* **2019**, *290*, 573-580.
3. Qian, Z.; Chai, L.; Tang, C.; Huang, Y.; Chen, J.; Feng, H. A Fluorometric Assay for Acetylcholinesterase Activity and Inhibitor Screening with Carbon Quantum Dots. *Sens. Actuators B Chem.* **2016**, *222*, 879-886.
4. Wang, M.; Gu, X.; Zhang, G.; Zhang, D.; Zhu, D. Continuous Colorimetric Assay for Acetylcholinesterase and Inhibitor Screening with Gold Nanoparticles. *Langmuir* **2009**, *25*, 2504-2507.
5. Xue, W.; Zhang, D.; Zhang, G.; Zhu, D. Colorimetric detection of Glucose and an Assay for Acetylcholinesterase with Amine-terminated Polydiacetylene Vesicles. *Sci. Bull.* **2011**, *56*, 1877-1883.
6. Ren, L.; Li, H.; Liu, M.; Du, J. Light-accelerating Oxidase-mimicking Activity of Black Phosphorus Quantum Dots for Colorimetric detection of Acetylcholinesterase Activity and Inhibitor Screening. *Analyst* **2020**, *145*, 8022-8029.
7. Lu, L.; Hu, X.; Zeng, R.; Lin, Q.; Huang, X.; Li, M.; Tang, D. Dual-mode Colorimetric-Photothermal Sensing Platform of Acetylcholinesterase Activity based on the Peroxidase-like Activity of Fe–N–C Nanozyme. *Anal. Chim. Acta* **2022**, *1229*, 340383.
8. Han, T.; Wang, G. Peroxidase-like Activity of Acetylcholine-based Colorimetric detection of Acetylcholinesterase Activity and an Organophosphorus Inhibitor. *J. Mater. Chem. B* **2019**, *7*, 2613-2618.
9. Du, D.; Huang, X.; Cai, J.; Zhang, A.; Ding, J.; Chen, S. An Amperometric Acetylthiocholine Sensor based on Immobilization of Acetylcholinesterase on a Multiwall Carbon Nanotube–Cross-linked Chitosan Composite. *Anal. Bioanal. Chem.* **2007**, *387*, 1059-1065.
10. Uchida, K.; Duenas, L.; Gomez, F. A. Thread- and Capillary Tube-Based Electrodes for the Detection of Glucose and Acetylthiocholine. *Micromachines* **2020**, *11*, 920.
11. Guan, J.; Wang, M.; Ma, R.; Liu, Q.; Sun, X.; Xiong, Y.; Chen, X. Single-atom Rh Nanozyme: An Efficient Catalyst for Highly Sensitive Colorimetric Detection of Acetylcholinesterase activity and Adrenaline. *Sens. Actuators B Chem.* **2023**, *375*, 132972.
12. Ying, M.; Yang, G.; Xu, Y.; Ye, H.; Lin, X.; Lu, Y.; Pan, H.; Bai, Y.; Du, M. Copper Fumarate with High-bifunctional Nanozyme activities at different pH values for Glucose and Epinephrine Colorimetric detection in Human Serum. *Analyst* **2022**, *147*, 40-47.
13. Guan, M.; Wang, M.; Qi, W.; Su, R.; He, Z. Biomineralization-inspired Copper-Cystine Nanoleaves Capable of Laccase-like Catalysis for the Colorimetric detection of Epinephrine. *Front. Chem. Sci. Eng.* **2021**, *15*, 310-318.
14. Maity, T.; Jain, S.; Solra, M.; Barman, S.; Rana, S. Robust and Reusable Laccase Mimetic Copper Oxide Nanozyme for Phenolic Oxidation and Biosensing. *ACS Sustain. Chem. Eng.* **2022**, *10*, 1398–1407.
15. Alizadeh, N.; Ghasemi, S.; Salimi, A.; Sham, T.-K.; Hallaj, R., CuO nanorods as a Laccase mimicking enzyme for highly sensitive Colorimetric and Electrochemical Dual Biosensor: Application in living cell epinephrine analysis. *Colloids Surf. B.* **2020**, *195*, 111228.
16. Godoy-Reyes, T.M.; Costero, A.M.; Gaviña, P.; Martínez-Máñez, R.; Sancenón, F. A Colorimetric Probe for the Selective Detection of Norepinephrine Based on a Double Molecular Recognition with Functionalized Gold Nanoparticles. *ACS Appl. Nano Mater.* **2019**, *2*, 1367-1373.
17. Menon, S.; Jose, A.R.; Jesny, S.; Kumar, K.G. A Colorimetric and Fluorometric Sensor for the Determination of Norepinephrine. *Anal. Methods.* **2016**, *8*, 5801-5805.
18. Ally, N.; Hendricks, N.; Gumbi, B. A Colorimetric Detection of Noradrenaline in Wastewater Using Citrate-Capped Colloidal Gold Nanoparticles Probe. *Colloids Inter.* **2022,** *6*, 61.
19. Son, S.E.; Ko, E.; Tran, V.-K.; Hur, W.; Choi, H.; Lee, H.B.; Park, Y.; Seong, G.H. Highly Sensitive Electrochemical Determination of Norepinephrine Using Poly Acrylic Acid-Coated Nanoceria. *ChemElectroChem.* **2019**, *6*, 4666-4673.
